# Economic evaluation in cardiac electrophysiology: Determining the value of emerging technologies

**DOI:** 10.3389/fcvm.2023.1142429

**Published:** 2023-04-26

**Authors:** Waseem Hijazi, Bert Vandenberk, Elissa Rennert-May, Amity Quinn, Glen Sumner, Derek S. Chew

**Affiliations:** ^1^ Libin Cardiovascular Institute, Department of Cardiac Sciences, University of Calgary, Calgary, AB, Canada; ^2^ Department of Cardiovascular Sciences, University of Leuven, Leuven, Belgium; ^3^ O’Brien Institute for Public Health, University of Calgary, Calgary, AB, Canada; ^4^ Department of Medicine, University of Calgary, Calgary, AB, Canada

**Keywords:** cost effectiveness analysis (CEA), economic evaluation (cost effectiveness), cardiac electrophysiogy, atrial fibrilation (AF), ICD (implantable cardioverter-defibrillator), CRT-D, cardiac resynchronization therapy–defibrillator, anticoagulation (AC), ablation < electrophysiology

## Abstract

Cardiac electrophysiology is a constantly evolving speciality that has benefited from technological innovation and refinements over the past several decades. Despite the potential of these technologies to reshape patient care, their upfront costs pose a challenge to health policymakers who are responsible for the assessment of the novel technology in the context of increasingly limited resources. In this context, it is critical for new therapies or technologies to demonstrate that the measured improvement in patients' outcomes for the cost of achieving that improvement is within conventional benchmarks for acceptable health care value. The field of Health Economics, specifically economic evaluation methods, facilitates this assessment of value in health care. In this review, we provide an overview of the basic principles of economic evaluation and provide historical applications within the field of cardiac electrophysiology. Specifically, the cost-effectiveness of catheter ablation for both atrial fibrillation (AF) and ventricular tachycardia, novel oral anticoagulants for stroke prevention in AF, left atrial appendage occlusion devices, implantable cardioverter defibrillators, and cardiac resynchronization therapy will be reviewed.

## Introduction

Cardiac electrophysiology has undergone substantial innovation over the past several decades. From the advent of leadless pacemakers and physiologic pacing (1–3) to newer catheter ablation techniques that use cryoablation and electroporation (4), electrophysiology is expanding rapidly with cutting-edge clinical technologies. Despite the potential of these technologies to reshape patient care, their upfront costs garner criticism from health policymakers who are responsible for the assessment of the novel technology in the context of increasingly limited resources. For instance, in the United States, health care costs as a proportion of the economy have risen dramatically over time. They now represent 19.7% of gross domestic product (GDP), up from 5.0% of GDP in 1960 (5). The European Union has seen healthcare costs per capita rise between 2012 and 2019, exceeding growth rates of gross domestic product per capita (6).

**Figure 1:**
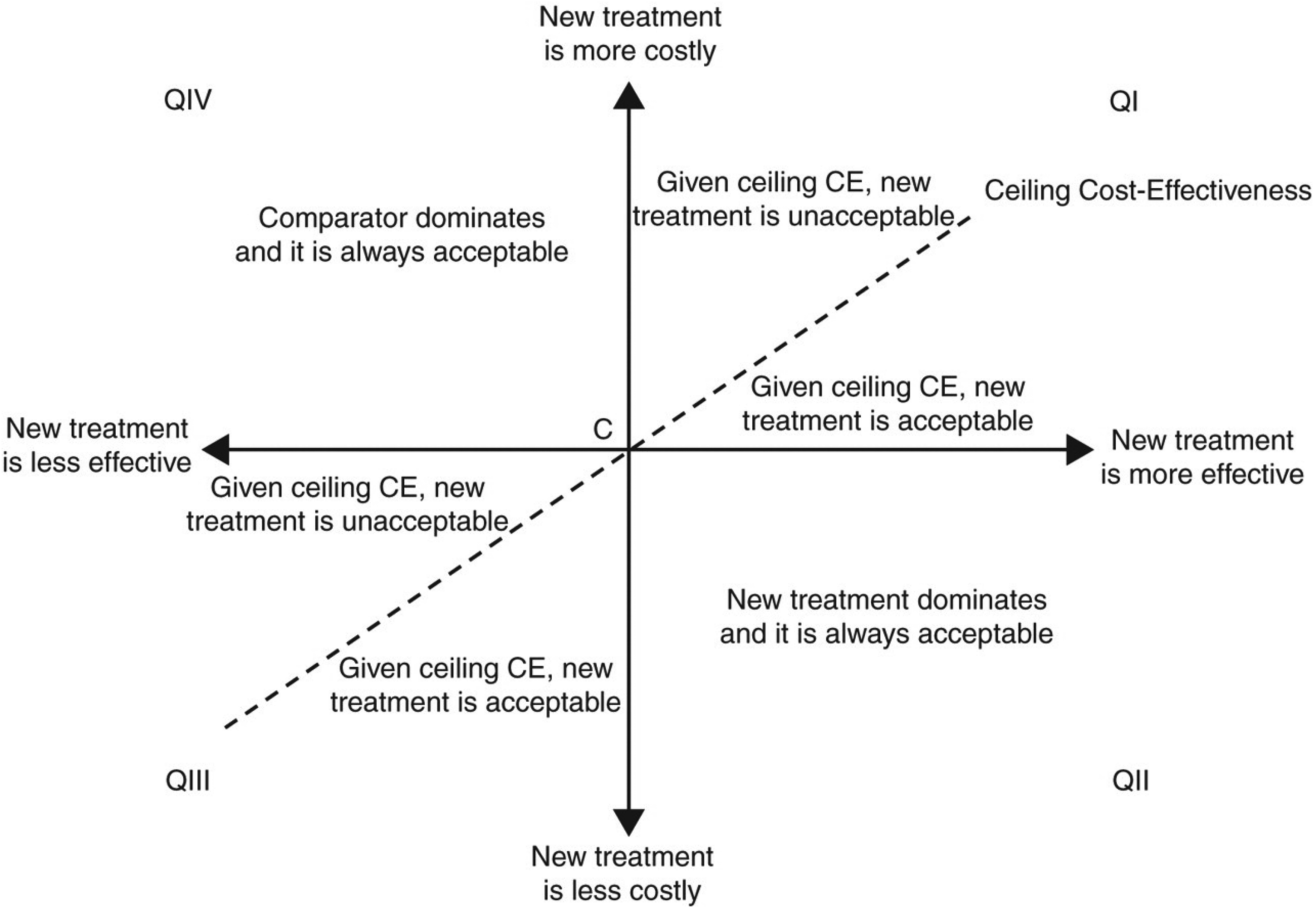
Cost-effectiveness plane. The horizontal axis represents the difference in benefits between the two therapies (e.g., difference in quality-adjusted life expectancy) and the vertical axis represents the difference in the cost. Quadrant II of the plane represents a situation where a new treatment “dominates” or is always acceptable than the comparator treatment; it reflects a situation where the new therapy is less costly and more effective. The opposite occurs in Quadrant IV, where the existing therapy dominates the new treatment. Quadrant I is where a new therapy is both more effective and most costly. If the incremental benefits are attained for acceptable incremental costs (this relationship is represented by the slope of the dotted line), then the new treatment is acceptable. [Reproduced from *EP Europace 2011; 13(Suppl_2):ii3–ii8* with permission].

In this context, it is critical for new therapies or technologies to demonstrate that the measured improvement in patients' outcomes for the cost of achieving that improvement is within conventional benchmarks for acceptable health care value. The field of Health Economics, specifically economic evaluation methods, facilitates this assessment of value in health care.

This review will provide an overview of economic evaluation to assess value in healthcare and discuss selected historical applications within cardiac electrophysiology. Specifically, catheter ablation for both atrial fibrillation (AF) and ventricular tachycardia (VT), novel oral anticoagulant agents for stroke prevention in AF, left atrial appendage occlusion (LAAO) devices, implantable cardioverter defibrillators (ICD), and cardiac resynchronization therapy (CRT) will be reviewed.

## Economic evaluation: a primer

Health policymakers are faced with decisions that pertain to which new therapies or technologies to adopt. In the field of health economics, these challenges are related to the fundamental principle of “opportunity cost,” which is defined as the potential benefits lost or forgone from other alternatives by choosing one alternative (7). Using leadless pacemakers as an example in the Norwegian Health System, Fagerlund and colleagues estimated that adoption of the Micra pacemaker over traditional transvenous pacemakers in 80 patients at high risk for complication from transvenous pacemaker implantation would require approximate 5 million Norwegian Krone per year (approximately 0.5 million US Dollars). If holding the health budget constant, adoption of the Micra pacemaker would require decreased funding elsewhere in the health budget, or more broadly, decreasing the budget elsewhere in the social sector to increase healthcare sector spending (8).

To help facilitate health policy decisions, economic evaluation is a field that assesses the “value” of a new technology or therapy. That is, such an evaluation poses the question: does a new technology represent good value for money? Does a new therapy provide additional benefits compared to conventional care for a reasonable price? The relationship between benefits and costs can be summarized as an incremental cost effectiveness ratio (ICER) (9, 10).

$$\mathrm{ICER}=\frac{\mathrm{Cost}\;A-\mathrm{Cost}\;B}{\mathrm{Effect}\;A-\mathrm{Effect}\;B}$$

For value comparisons across medicine, the ICER is most commonly assigned the units of a cost ($) per quality-adjusted life year (QALY) gained. QALYs represent patient life expectancy adjusted by quality-of-life. That is, each year of life expectancy is scaled by the quality of life experienced by the patient for a given disease, e.g., heart failure. This scaling factor, also known as a utility, can range from 0 to 1, where 1 denotes perfect health and 0 denotes death (11).

The relationship between incremental costs (Cost A − Cost B) and incremental clinical benefits (Effect A − Effect B) is shown above. An ideal scenario would be to adopt a new therapy, which is both more effective than its comparator and less costly. However, few technologies in cardiac sciences are truly *cost-savings*. Most new therapies provide additional clinical benefits for increased costs (Figure 1). A recent example in the area of heart failure is dapagliflozin, which conferred clinical benefits (i.e., reduced risk of HF hospitalization and death from cardiovascular causes) in symptomatic heart failure (HF) patients (12, 13). In a cost-effectiveness analysis of the landmark DAPA-HF (Dapagliflozin and Prevention of Adverse Outcomes in Heart Failure) trial, patients in the dapagliflozin arm accrued an additional 0.46 QALYs compared to placebo for an additional cost of $38,212, which included $35,708 in dapagliflozin costs over a patient's lifetime horizon (14). The “time horizon” describes the timeframe over which these cost and benefits are calculated. Economic evaluation in cardiology often adopts a lifetime horizon, given the chronic nature of the disease processes, to ensure that all relevant costs and benefits are captured.

Whether a therapy is considered cost-effective depends on country-specific thresholds for value in healthcare. ICERs that fall below these willingness-to-pay thresholds are considered economically attractive. The World Health Organization has suggested an approximate benchmark of 3 times the gross domestic product per capita as an upper threshold for acceptable cost-effectiveness for a given country (15, 16). However, this definition is not routinely used by national health technology assessment agencies. In Canada and Europe, conventional thresholds range from $50,000 to $100,000 per QALY gained or £20,000–30,000, respectively (17, 18). In the United States, a value taxonomy has been proposed by the American College of Cardiology and American Heart Association where high-value represents either cost-savings or an ICER <$50,000 per QALY gained, intermediate value is represented by ICERs between $50,000 to <$150,000 per QALY gained, and low value is described by ICERs ≥$150,000 per QALY gained (19).

### Types of economic evaluation

Economic evaluation can take several forms (Table 1). Fundamentally, all these types of economic evaluation assess how a particular intervention influences costs and benefits. Cost-effectiveness (CEA) and cost-utility (CUA) analyses are the most common types of economic evaluation, which are used to derive an ICER comparing two therapies. In CEA, benefits are commonly expressed in natural units such as life expectancy or life-years (LYs) gained. The “natural units” may also be expressed as disease-specific, clinically relevant endpoints. For example, in a CEA of an antibacterial eluting envelop to reduce post-operative infections associated with cardiac implantable electronic device implantation, the ICER was expressed as a cost per infection prevented (20). However, the use of natural units other than life expectancy limits value comparison across diseases.

**Table 1:** Key methodologies in health economic evaluation.

Methodology	Key Aspects
Cost-Effectiveness Analysis (CEA)	• Compares costs with incremental clinical benefits expressed in “natural units”, often life-years • ICER usually expressed in $/LY
Cost-Utility Analysis (CUA)	• Benefits are valued in terms of life expectancy and quality of life • ICER expressed in $/QALY
Cost-Benefit Analysis (CBA)	• Costs and benefits both measured in monetary units • Allows direct cost comparisons but lacks transparency in the relationship of clinical outcomes as these are converted to costs
Cost-Minimization Analysis (CMA)	• Assumes equal benefit of both interventions and compares costs only • Aim is to choose the least expensive option

A cost-utility analysis is similar to a CEA, but also incorporates quality of life into the measure of clinical benefit. CUAs facilitate comparisons across different interventions and disease states by using a common standard of clinical benefits (i.e., QALYs). Other forms of economic evaluation include cost-minimization analysis, which considers the least costly alternative and assumes that the benefits/outcomes of two therapies are identical, and cost-benefit analysis, where both the costs and benefits of a therapy are expressed in monetary terms (21).

## Applied health economics in cardiac electrophysiology

### Catheter ablation for atrial fibrillation

Catheter ablation is an established therapy for patients with symptomatic atrial fibrillation. It has been shown to reduce AF recurrences, provide durable improvements in quality of life compared to medical therapy alone, and attenuate progression of the natural history of AF (22–25). Furthermore, among patients with impaired left ventricular function, catheter ablation improves survival and reduces HF hospitalization (26).

However, in patients where a rhythm control strategy is clinically appropriate, the upfront costs of catheter ablation are noticeably greater than an antiarrhythmic medication approach, due to the human and material costs of the procedure, as well as the outpatient diagnostic testing for follow-up and peri-operative imaging that may be required. The cost of catheter ablation procedure varies depending on the country and regional context; the average cost per patient is estimated to be approximately $27,000–$38,000 (2021 USD) in the United States (27, 28), $15,000 in Canada (2021 CAD) (29–31), and £7,000 (2021 GBP) (32). In comparison, medical therapy is estimated to cost less than a quarter of overall ablation costs annually (30).

While these upfront costs are substantive, cost-effectiveness analyses have attempted to provide a more comprehensive assessment of costs relative to clinical benefits projected over a longer follow up duration. That is, after ablation there may be cost offsets from the reduction in symptomatic AF burden and potentially averted HF hospitalizations. Further, there may be fewer drug complications after discontinuation of long-term antiarrhythmic medications (33). These favourable outcomes may contribute to a sustained improvement in quality of life for people with AF.

Early cost effectiveness analyses using modelling-based approaches showed that catheter ablation of AF was economically attractive compared to drug therapy alone (Table 2). For example, a Canadian analysis demonstrated an ICER of $59,194 per QALY gained for catheter ablation compared to amiodarone using a 5-year time horizon in a population of symptomatic patients on first-line anti-arrhythmic drug therapy (35). A British analysis in a similar population also demonstrated an ICER of £7,763 per QALY gained (34). However, early analyses were limited due to modelling assumptions that catheter ablation reduced stroke risk, based on the prevailing assumption at the time where rhythm control conferred reduced stroke risk (39). Additionally, these analyses did not have the benefit of longer-term clinical effectiveness data reporting hard cardiovascular outcomes.

**Table 2:** Selected economic evaluations of catheter ablation for atrial fibrillation.

Study (Year)	Patient population	Analysis/Methodology	Country	Intervention vs. Comparator	Time Horizon	Currency	Incremental Cost Effectiveness Ratio	Reference
McKenna (2009)	Patients with paroxysmal AF refractory to medical therapy	CUA/Markov model	United Kingdom	Catheter ablation vs. AAD	Lifetime	2006 GBP	£7763–£7910/QALY	(34)
Blackhouse (2013)	Patients with drug-refractory AF	CUA/Markov model	United States	Catheter ablation vs. amiodarone	5 years	2010 CDN	$59,194/QALY	(35)
Aronsson (2015)	Patients with symptomatic, antiarrhythmic drug naïve AF within the preceding 6 months.	CUA/Markov model	Sweden	Catheter ablation vs. AAD	Lifetime	2012 Euros	€50,570/QALY	(36)
Chew (2022)	Patients with paroxysmal or persistent AF aged ≥65 years or <65 years with ≥1 risk factors for stroke	CUA/Trial-based	United States	Catheter ablation vs. medical therapy	Lifetime	2018 USD	$57,893/QALY $183,318/LY	(28)
**Catheter ablation in Atrial Fibrillation and Heart Failure**								
Gao (2019)	Patients with concomitant symptomatic AF and HF with reduced EF	CEA and CUA/ Markov model	Australia	Catheter ablation vs. medical therapy	Lifetime	AUD	$55,942/QALY $35,020/LY	(37)
Chew (2020)	Patients with concomitant symptomatic AF and HF with reduced EF	CUA/Markov model	United States	Catheter ablation vs. medical therapy	Lifetime	2018 USD	$38,496/QALY	(38)
Lau (2021)	Patients with concomitant symptomatic AF and HF with reduced EF	CUA/Markov model	Canada	Catheter ablation vs. medical therapy	Lifetime	2018 CDN	$35,360/QALY	(30)

AAD, antiarrhythmic drug; AF, atrial fibrillation; AUD, Australian Dollar; CDN, Canadian Dollar; CUA, cost utility analysis; GBP, British Pound Sterling; EF, ejection fraction; HF, heart failure; QALY, quality adjusted life years; LY, life years; USD, US Dollar.

More recently, the CABANA (Catheter ABlation vs. ANtiarrhythmic Drug Therapy for Atrial Fibrillation) study, the largest clinical trial on catheter ablation for AF vs. pharmacotherapy, did not find a difference in the primary composite endpoint of death, stroke, serious bleeding and cardiac arrest hazard ratio (HR) 0.86 [95% confidence interval (CI) 0.65–1.15] (22). However, the trial demonstrated improved quality of life and less AF recurrence with catheter ablation.

In the economic substudy of CABANA conducted from the U.S. healthcare perspective, catheter ablation was associated with an ICER of $57,893 per QALY gained compared to drug therapy alone, which falls within conventional U.S. societal thresholds for good value in health care. Generally speaking, cost-utility analyses summarize clinical benefits as a QALY. Thus, the clinical benefits that may drive cost-effectiveness are either survival, quality of life or both in some combination. Since CABANA did not demonstrate improved survival among individuals with AF randomized to catheter ablation in the intention-to-treat analysis, cost-effectiveness was contingent solely on quality-of-life gains. Note, that without quality-of-life adjustments, the ICER was $183,318 per LY gained (28).

However, emerging evidence suggests that certain subgroups with AF may also derive mortality benefit from catheter ablation compared to medical therapy in addition to increased quality of life. In these subgroups, one would anticipate an even more attractive value proposition as cost-effectiveness would be motivated by increases in life expectancy as well as quality of life. For example, in the CASTLE AF (Catheter Ablation for Atrial Fibrillation with Heart Failure) trial, catheter ablation prevented all-cause mortality relative to pharmacotherapy with a HR of 0.53 (95% CI: 0.32–0.86) (26) in patients with HF and reduced ejection fraction (EF ≤35%). In the HF subgroup of CABANA, which included 778 patients, demonstrated a 43% reduction in mortality in addition to quality-of-life improvements and freedom from AF recurrence. Of note, most patients in this analysis had heart failure with preserved ejection fraction (EF >50%) (40).

Accordingly, several cost-effectiveness analyses have noted catheter ablation to be economically attractive across a several country settings and in patients with either HF and reduced or preserved ejection fraction. For individuals with concomitant AF with HF reduced ejection fraction, the estimated ICERs were $35,360 per QALY gained in Canada (30), $35,020 per LY in Australia (37) and $38,496 per QALY gained in the United States (38). Among those with HF and preserved ejection fraction, there also appears to be an economic benefit conferred by catheter ablation of AF ($54,135 per QALY in the United States) (28).

In summary, catheter ablation for AF appears to be good “value” for money by improving quality of life and in some subsets, such as heart failure, offering mortality benefit in addition to quality-of-life gains. However, these benefits are accrued at increased costs over a patient's lifetime; despite studies suggesting reduced health resource use post-ablation, catheter ablation is not cost-savings overall (41, 42). Given an aging population and the rising prevalence of AF, increasing demand for catheter ablation highlights another important consideration in policy decision making—affordability. This concept is distinct from the economic concept of efficiency, or value for money, which is the focus of cost-effectiveness analyses. Nonetheless, the landscape of catheter ablation continues to evolve. Future work will be required to understand the economics of newer catheter technology, such as pulse field/electroporation and cryoballoon therapy (43), or providing catheter ablation of selected patient subgroups such as those early in the AF disease course, as suggested by EAST-AFNET-6 (Early Treatment of Atrial Fibrillation for Stroke Prevention Trial) and ablation trials such as EARLY AF (Early Aggressive Invasive Intervention for Atrial Fibrillation) and PROGRESSIVE AF (Impact of First-Line Rhythm Therapy on AF Progression) (25, 44, 45).

### Stroke and thromboembolism prevention in atrial fibrillation: anticoagulation

Anticoagulation is the mainstay for stroke and thromboembolism prevention in atrial fibrillation. Historically, despite the advantages over placebo, aspirin monotherapy, and combination antiplatelet therapy, clinical practice rates of warfarin among eligible patients remained suboptimal at below 60% (46, 47). Possible barriers to warfarin use in clinical practice include its narrow therapeutic window and requirement for ongoing dose adjustment and monitoring. The approval of direct oral anticoagulants (DOACs) over the past decade has offered a safe effective alternative, which represents a significant evolution in stroke prevention therapy (48, 49).

Since the cost of warfarin was much less expensive at the time of DOAC approval, DOACs required compelling additional clinical benefits to be considered cost-effective by conventional benchmarks for good value in health care. Indeed, an individual patient-level meta-analysis of the landmark DOAC trials showed that standard-dose DOACs, compared to warfarin, were associated with a significantly lower hazard of stroke or systemic embolism (HR: 0.81; 95% CI: 0.74–0.89), all-cause death (HR: 0.92; 95% CI: 0.87–0.97), and intracranial bleeding (HR: 0.45; 95% CI: 0.37–0.56) (50).

Accordingly, the majority of cost-effectiveness studies comparing individual DOACs to warfarin have estimated favorable ICERs that fall below country-specific willingness-to-pay thresholds (Table 3) (51–53). For example, in the trial-based economic evaluation of the ARISTOTLE (Apixaban for Reduction in Stroke and Other Thromboembolic Events in Atrial Fibrillation) trial, comparing apixaban to warfarin, the ICER was $53,825 per QALY gained from the US healthcare perspective (58). From the Belgian healthcare payer perspective, rivaroxaban was economically attractive compared to warfarin for stroke prevention in patients with atrial fibrillation. Using cohort level data from the ROCKET AF (Rivaroxaban Once daily oral direct factor Xa inhibition Compared with vitamin K antagonism for prevention of stroke and Embolism Trial in AF) trial to inform a Markov model, the estimated ICER was €8,809 per QALY and the probability of cost-effectiveness was 87% at a threshold of €35,000 per QALY gained (54).

**Table 3:** Selected economic evaluations of anticoagulation for stroke prevention in atrial fibrillation.

Study (Year)	Patient population	Analysis/Methodology	Country	Intervention vs. Comparator	Time Horizon	Currency	Incremental Cost Effectiveness Ratio	Reference
Freeman (2011)	Patients aged 65 years or older with nonvalvular AF and risk factors for stroke (CHADS_2_ score ≥1) and no contraindications to anticoagulation.	CUA/Markov model	United Kingdom	High-dose (150 mg bid) or low-dose (100 mg bid) dabigatran vs. warfarin	Lifetime	2008 US$	$45,372/QALY (High Dose Dabigatran) $51,229/QALY (Low Dose Dabigatran)	(51)
Kleintjens (2013)	Patients with non-valvular AF at moderate to high risk of stroke (CHADS_2_ score ≥2)	CUA/Markov model	Belgium	rivaroxaban vs. warfarin	Lifetime	2010 Euros	€7,493/LY €8,809/QALY	(54)
Canestaro (2013)	Patients 70 years or older with atrial fibrillation	CUA/Markov model	United States	dabigatran vs. rivaroxaban vs. apixaban vs. warfarin	Lifetime	2011 USD	Compared to warfarin: $93,063/QALY (apixaban) $111,465/QALY (rivaroxaban) $140,557/QALY (dabigatran)	(55)
You (2014)	Patients with AF at risk of stroke (CHADS_2_ scores ≥2)	CUA/Markov model	United States	DOACs (apixaban, dabigatran and rivaroxaban) vs. warfarin [stratified by time in therapeutic range (TTR)]	Lifetime	2013 USD	$35,804/QALY (60% TTR) $60,141/QALY (70% TTR) $79,268/QALY (75% TTR)	(56)
Shah (2016)	Patients with AF at risk of stroke	CUA/Markov model	United States	dabigatran vs. rivaroxaban vs. apixaban vs. edoxaban vs. warfarin	Lifetime	2015 USD	Compared to warfarin: $25,816/QALY (apixaban) $27,643/QALY (edoxaban) $57,434/QALY (rivaroxaban) $31,435/QALY (dabigatran)	(57)
Cowper (2017)	Patients with AF and 1 or more additional risk factors for stroke	CUA/Trial-based	United States	apixaban vs. warfarin	Lifetime	2014 USD	$53,925/QALY	(58)
Wu (2021)	Patients older than 75 years with AF	CUA/Markov model	United States	dabigatran vs. rivaroxaban vs. apixaban vs. edoxaban vs. warfarin	10 years	2020 USD	Compared to warfarin: $112,439/QALY (dabigatran) $71,587/QALY (rivaroxaban) $52,800/QALY (apixaban) $15,865/QALY (edoxaban)	(59)

AF, atrial fibrillation; CDN, Canadian Dollar; CUA, cost utility analysis; DOAC, direct oral anticoagulant; HF, heart failure; QALY, quality adjusted life years; LY, life years; TTR, time in therapeutic range; USD, US Dollar.

A recurring theme in the sensitivity analyses of these economic evaluations is the price of DOAC as a determinant of the ICER. That is, the greatest variation in cost-effectiveness is due to the initial DOAC price used to estimate the ICER. For example, in the cost-effectiveness analysis of the ARISTOTLE trial comparing apixaban to warfarin, the ICER decreased from $53,825 to $26,927 per QALY gained with a 50% reduction in apixaban price (58). With the imminent arrival of generic formulations of several DOACs, the value proposition is expected to improve. Nevertheless, even at current prices, DOACs are considered the standard of care from both an economic and clinical perspective across the major cardiovascular societies in North America and Europe (60–62).

In some health jurisdictions where health technology assessment is used to guide funding decisions, such as the United Kingdom, it will be particularly important that any new anticoagulant that comes to market demonstrates improved value compared to the existing standard of care (i.e., DOACs). This may be accomplished in several ways: (a) an improved safety profile, which may translate to less decrement in quality of life from bleeding events; (b) improved clinical effectiveness with additional stroke reduction, which would improve quality of life and possibly survival; and (c) comparable or lower price than DOACs, which is unlikely due to the impacts on return on investment. Recently, an oral factor Xia inhibitor has shown promise in a phase 2 dose finding study; the PACIFIC-AF (Safety of the oral factor XIa inhibitor asundexian compared with apixaban in patients with atrial fibrillation) trial recently showed that asundexian had a two-thirds reduction in bleeding risk of apixaban in patients with AF and stroke risk based on CHA_2_*DS*_2_-VASc score risk (63). Work is ongoing to confirm these findings in Phase III trials.

### Stroke prevention in atrial fibrillation: left atrial appendage occlusion

Left atrial appendage occlusion has been proposed as a non-pharmacologic strategy for stroke prevention in AF. The majority of clinical data supporting left atrial appendage closure comes from two seminal trials, PREVAIL (Evaluation of the WATCHMAN LAA Closure Device in Patients With Atrial Fibrillation Versus Long Term Warfarin Therapy) and PROTECT-AF (WATCHMAN Left Atrial Appendage System for Embolic Protection in Patients With Atrial Fibrillation) (64, 65). In patient-level meta-analysis that pooled the five-year outcome data from both trials, there were significant reductions in hemorrhagic stroke, cardiovascular death, all-cause death and post-procedure bleeding with LAA closure compared to warfarin anticoagulation (66).

However, there are ongoing concerns regarding the effectiveness of LAA closure regarding ischemic stroke and systemic embolism prevention. That is, the rate of ischemic stroke and systemic embolism was numerically higher with LAAO compared to warfarin in the meta-analysis, albeit these results were not statistically significant (hazard ratio 1.71; *p* = 0.080). Additionally, the PREVAIL trial failed to demonstrate non-inferiority of their coprimary composite endpoint of stroke, systemic embolism, or cardiovascular/unexplained death (65).

**Figure 2:**
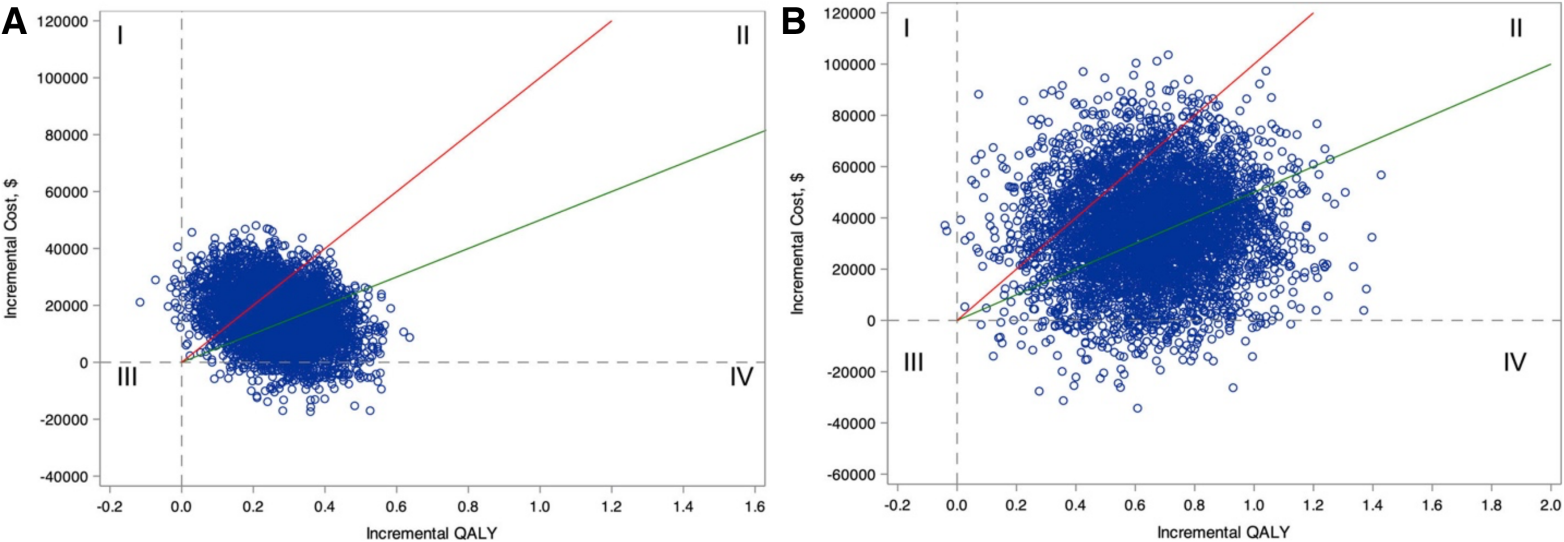
Incremental cost-effectiveness plane for comparing ablation with drug therapy for the entire CABANA cohort (catheter ablation vs. Antiarrhythmic Drug Therapy for Atrial Fibrillation; *N* = 2204; **A**) and the heart failure subgroup with New York Heart Association class ≥II symptoms (*n* = 778; **B**). Estimates of incremental costs and quality-adjusted life-years (QALYs) are shown (1 blue circle for each of 5,000 bootstrap samples). Quadrant I represents scenarios where ablation is more costly and less effective, Quadrant II represents scenarios where ablation is more costly and effective, Quadrant III represents scenarios where ablation is less costly and less effective, and Quadrant IV represents scenarios where ablation is less costly and more effective. The willingness-to-pay thresholds of $50,000 and $100,000 per QALY gained are represented as the slope of the green and red lines, respectively. Scenarios that fall below these willingness-to-pay-thresholds are considered economically attractive. (Reproduced from *Circulation. 2022;146:535–547* with permission).

Nevertheless, an advantage to health economics methods is the ability to quantify the uncertainty in estimates of projected benefit, costs, and cost-effectiveness. Using probabilistic sensitivity analysis, each model input incorporates the surrounding confidence interval. Each time the economic model is executed, a single estimated ICER is generated by sampling inputs from their respective distributions rather than using mean parameter value. The model is then repeated many times (e.g., 1,000 or 10,000 simulations) to estimate the probability that the ICER meets benchmarks for cost-effectiveness (Figure 2).

Applying probabilistic sensitivity analyses in the cost-effectiveness analysis of the pooled PREVAIL and PROTECT AF trials, Reddy and colleagues found that LAAO was economically attractive compared to warfarin in the US setting with an estimated ICER of $48,674 per QALY by year 7 post implant, and cost-savings by year 10 (67). In the probabilistic sensitivity analysis with 10,000 simulations, the probability of LAAO cost-effectiveness was 98% when compared to warfarin.

One important caveat is that majority of these LAAO cost-effectiveness studies rely on clinical effectiveness data that compare a single LAAO device, Watchman (Boston Scientific) to warfarin. There is limited generalizability to other LAAO devices, such as the Amplatzer Amulet (Abbott), due to differences in upfront device costs, complications, and long-term effectiveness. Additionally, with improved safety profile of DOACs relative to warfarin, the relative advantage of LAAOs on bleeding risk becomes less certain (68). One analysis in the Canadian context suggested that DOACs were more cost-effective than LAAO occlusion with apixaban dominating dabigatran, LAA occlusion and rivaroxaban (69). However, there is limited data on the comparative efficacy of DOACs vs. LAAO to inform the inputs of current modelling studies, limiting their application to health policy decision making.

Future cost-effectiveness studies will benefit from upcoming clinical trials comparing LAAO to DOACs including CATALYST (Clinical Trial of Atrial Fibrillation Patients Comparing Left Atrial Appendage Occlusion Therapy to Non-vitamin K Antagonist Oral Anticoagulants; ClinicalTrials.gov NCT04226547), CHAMPION-AF (WATCHMAN FLX Versus NOAC for Embolic Protection in in the Management of Patients With Non-Valvular Atrial Fibrillation; ClinicalTrials.gov NCT04394546), and Occlusion-AF (Left Atrial Appendage Occlusion Versus Novel Oral Anticoagulation for Stroke Prevention in Atrial Fibrillation; ClinicalTrials.gov NCT03642509).

### Implantable-cardioverter defibrillators and cardiac resynchronization therapy

Sudden cardiac death is estimated to account for up to 20% of global mortality, and prevention and treatment of sudden cardiac death remains a significant public health challenge (70). In the 1990s, ICDs emerged as a disruptive technology for prevention of sudden death. ICDs were initially used in a relatively limited setting for secondary prevention among patients who had been resuscitated from malignant ventricular arrhythmias. However, the majority of sudden cardiac deaths are sustained among patients without prior episodes of sustained VT or ventricular fibrillation (70).

The evidence for ICD use in primary prevention populations (i.e., patients at risk for sudden death, but no history of sustained ventricular arrhythmias) were largely informed by two large randomized trials, SCD-HeFT (Sudden Cardiac Death in Heart Failure Trial) and MADIT II (Multicenter Automatic Defibrillator Implantation Trial II), which showed substantial improvements in all-cause survival with the ICD therapy (71, 72). These expanded indications for ICDs posed a challenged to health policymakers due to the opportunity cost associated with offering ICD implantation in a larger pool of eligibility patients (73, 74). An estimated 100,000 patients receive an ICD annually in the United States (75), although this number is an underestimate of the eligible population; <50% of the eligible individuals actually receive an ICD (76, 77). Although the reasons are multifactorial and complex, the low ICD uptake was initially thought in part due to the high cost of ICDs and anticipated economic burden on healthcare budgets (73). In the 2000s, US acquisition prices for the device alone ranged from $22,000 to $52,000 (reported in 2022 USD) (73)—not including the human and infrastructural resources required to implant the device and follow up patients in the long-term (78).

High-quality cost-effectiveness analyses of ICDs conducted in a variety of geographic settings estimate ICERs comparable with other well-accepted therapies despite high lifetime costs associated with ICD therapy (Table 4) (79–82). A notable feature of these cost-effectiveness studies is that the majority of lifetime costs occur upfront at the time of ICD implantation, while benefits take years to accrue. The influence of delayed clinical benefit is best demonstrated in the economic sub-study of the SCD-HeFT trial. Mark et al*.* found that primary prevention ICD was economically attractive compared to amiodarone (ICER of $38,389 per LY gained), but this finding was dependent on survival past the five-year follow-up of the SCD-HeFT trial. Indeed, at 5 years post-implantation, the ICER for primary prevention ICD was $127,503/LY. This later fell to $88,657/LY at 8 years of follow-up (79). Current best practice guidelines for economic evaluation recommend choosing a time horizon where all relevant costs and benefits are captured (87). In the case of primary prevention ICD implantation, these benefits are expected to accrue over a patient's lifetime, in which case a lifetime horizon or follow up is appropriate.

**Table 4:** Selected economic evaluations of implantable cardioverter defibrillator and cardiac resynchronization therapy devices.

Study (Year)	Patient population	Analysis/Methodology	Country	Intervention vs. Comparator	Time Horizon	Currency	Incremental Cost Effectiveness Ratio	Reference
**Implantable Cardioverter Defibrillators—Primary Prevention**								
Sanders (2005)	Primary prevention device in LV dysfunction	CEA and CUA/Markov decision model	United States	ICD vs. Medical therapy	Lifetime	2005 USD	$24,500–$50,700/LY $34,000–$70,200/QALY	(80)
Mark (2006)	Primary Prevention; Patients with LV dysfunction with EF <35%, NYHA Class II-III	CEA and CUA/Trial-based	United States	ICD vs. Medical therapy	Lifetime	2003 USD	$38,389/LY $41,530/QALY	(79)
Smith (2013)	Primary Prevention; Patients with LV dysfunction, EF <40%	CUA/Markov decision model	United Kingdom	ICD vs. no ICD	Lifetime	2010 Euro	€43,993/QALY	(82)
**Implantable Cardioverter Defibrillators—Secondary Prevention**								
O’Brien (2001)	Secondary prevention; Patients surviving VT/VF	CEA/Trial-based	Canada	ICD vs. amiodarone	6 years	1999 CDN	$214,543/LY	(78)
Larsen (2002)	Secondary prevention; Patients resuscitated from cardiac arrest or ventricular tachycardia causing syncope or severe hemodynamic impairment and EF ≤0.40	CEA/Trial-based	United States	ICD vs. antiarrhythmic therapy	4 years	1997 USD	$66,677/LY	(83)
**Cardiac Resynchronization Therapy**								
Feldman (2005)	Patients with symptomatic HF (NYHA class III/IV), EF ≤35%, QRS ≥120 ms, PR >150 ms, HF hospitalization within 1 year	CUA/Markov decision model	United States	CRT-P or CRT-D vs. medical therapy	7 years	2004 USD	$19,600/QALY (CRT-P) $43,000/QALY (CRT-D)	(84)
Noyes (2013)	Minimally symptomatic (NYHA I/II)HF, QRS >130 ms, EF <30%	CUA/Trial-based	United States	CRT vs. ICD	4 years	2008 USD	$58,330/QALY (full cohort) $16,640/QALY (LBBB subgroup)	(85)
Woo (2015)	Patients with mild HF (NYHA class I/II), QRS >120 ms, EF <30%	CUA/Markov decision model	United States	CRT-D vs. ICD alone	Lifetime	2014 USD	$61,700/QALY	(86)

CDN, Canadian; CRT-D, cardiac resynchronization therapy defibrillator; CRT-P, cardiac resynchronization therapy pacemaker; CUA, cost utility analysis; ICD, implantable cardioverter defibrillator; EF, ejection fraction; HF, heart failure; QALY, quality adjusted life years; LV, left ventricular; LY, life years; NYHA, New York Heart Association; VF, ventricular fibrillation; VT, ventricular tachycardia.

Following the development of CRT systems, there were similar concerns among healthcare administrators regarding increasing costs of medical technology as a proportion of healthcare spending (88). These financial concerns, such as budget limitations and differences in reimbursement, may in part explain higher CRT implantation rates in the US compared to Europe, and the variation among European countries themselves (88). Nevertheless, the use of CRT plus an ICD among patients with severe LV systolic dysfunction and evidence of dyssynchrony [i.e., left bundle branch block (LBBB)] is cost-effective beyond ICD therapy alone. That is, the addition of a coronary sinus lead provides additional mortality and morbidity benefits, as well as improved quality of life, for additional costs within conventional thresholds for healthcare value (85, 86). For example, Mealing and colleagues performed a complex decision analytic model from the perspective of the United Kingdom National Health System comparing ICDs, CRT-pacemakers and CRT-defibrillators (CRT-D) informed by pooled individual patient data from 13 randomized clinical trials (89). Among patients with EF ≤35% and LBBB, CRT-D was considered cost-effective compared to ICD therapy alone at a willingness to pay threshold of £30,000 per QALY gained. However, with more severe heart failure symptoms [i.e., New York Heart Association (NYHA) class III or IV], CRT-P was also considered cost-effective relative to either CRT-D therapy or ICD/medical therapy alone.

There are several factors that may influence the cost-effectiveness of device therapy therapy in the contemporary context. For example, the cost of an CRT ± ICD has decreased with free market competition (90) and improved technology, specifically improved battery life has reduced the frequency of generator replacements (91). However, efficiencies gained through lower incremental costs are tempered by possible attenuation of clinical benefit in among subgroups of candidate patients due to the evolution in prerequisite guideline-directed medical therapy. For example, the absolute risk, and thereby the absolute risk reduction in mortality conferred by ICD therapy, is decreased with improvements in medical therapy for left ventricular dysfunction. An analysis of randomized clinical trials enrolling patients with heart failure and reduced ejection fraction between 1995 and 2014 showed there was a 44% decline in the rate of sudden death over time (92). Furthermore, the benefit of primary prevention ICDs among patients with non-ischemic etiology has become more controversial upon publication of the DANISH (Danish Study to Assess the Efficacy of ICDs in Patients with Non-ischemic Systolic Heart Failure on Mortality) trial, which found no benefit to ICD therapy compared to standard medical therapy in patients with an EF less than 35% and no coronary disease (HR: 0.87; 95% CI: 0.68–1.12) (93). Compared to previous trials which also enrolled patients with non-ischemic cardiomyopathy (94), DANISH enrolled a patient cohort from a decade later, who were optimized on more contemporary medical therapy (i.e., higher use of mineralocorticoid antagonists) and who had a higher proportion of CRT device use (93).

In summary, the value proposition of medical devices is not static. Updated cost-effectiveness analyses are necessary to account for changing conditions that influence the value of a given therapy. In the case of an ICD, future cost-effectiveness analysis should account for decreased ICD acquisition costs, decreased costs from less frequent generator replacements with improved ICD battery technology, contemporary device programming, and the influence of the current guideline-directed medical therapies on baseline sudden cardiac death risk. Furthermore, economic studies are required to evaluate newer, more expensive ICD technology, such as subcutaneous ICDs, compared to the current standard of transvenous ICDs.

Similar to ICDs, there may be a shift in the value proposition of CRTs. The relatively recent development of physiologic pacing techniques, such as His bundle or left bundle branch area pacing, offers the potential for similar resynchronization benefits to CRTs for decreased implantation costs (95, 96). Large clinical trials are currently underway assessing the clinical effectiveness of traditional CRT to physiologic pacing, such as the Left vs. Left trial (Cardiac Resynchronization Therapy Using His/Left Bundle Pacing vs. Left Ventricular Epicardial Pacing in Patients with Heart Failure; ClinicalTrials.gov NCT05650658), which aim to enroll 2,139 participants to compare His or Left bundle branch area pacing vs. biventricular pacing in patients with heart failure due to left ventricular systolic dysfunction (EF ≤50%) and with either a wide QRS (≥130 ms) or >40% pacing optimized on guideline-directed medical therapy.

### Catheter ablation for ventricular tachycardia in the setting of structural heart disease

Multiple randomized clinical trials have shown that catheter ablation is an important treatment for VT particularly in the context of ischemic cardiomyopathy. A recent meta-analysis of nine trials comparing ablation to antiarrhythmic therapy in patients with structural heart disease and VT concluded that ablation reduced the risk of VT recurrence and ICD therapies, but had no effect on heart failure hospitalization, cardiovascular mortality or all-cause mortality (97).

VT catheter ablation can be a time intensive and complex procedure associated with substantial upfront costs. Unlike catheter ablation for AF, foci causing VT in the context of cardiomyopathy can be endocardial or epicardial, and in many different anatomical locations. Substrate mapping is often long and requires highly specialized operators and equipment. Thus, a focus on cost-effectiveness is of increasing importance for this procedure to assess the relative balance between upfront costs, variable effect on patient outcomes (i.e., improved quality of life, reduced ICD shocks but no difference in mortality), and downstream cost reduction from decreased medical resource use associated with recurrent VT events and ICD shocks (98).

There is very limited data on the cost-effectiveness of VT ablation in cardiomyopathy (Table 5). One cost effectiveness analysis conducted from the UK perspective compared VT catheter ablation to anti-arrhythmic therapy among patients with ischemic cardiomyopathy and an ICD (100). The study found that catheter ablation was unlike to be cost-effective with an estimated ICER of £144,150 per quality-adjusted life-year gained, over a 5-year time horizon, which falls outside the UK's willingness to pay thresholds for value in healthcare. Consistent with the available clinical trials at the time, the benefit of ablation was driven by small gains in quality of life, but not mortality. However, a limitation noted by the authors was the lack of robust trial data reporting quality of life; only three of six trials that informed the analysis infrequently measured health-related quality of life (100).

**Table 5:** Selected economic evaluations of catheter ablation for ventricular tachycardia in the setting of structural heart disease.

Study (Year)	Patient population	Analysis/Methodology	Country	Intervention vs. Comparator	Time Horizon	Currency	Incremental Cost Effectiveness Ratio	Reference
Coyle (2018)	Patients with ICDs and ischemic cardiomyopathy with drug-refractory VT/VF	CUA/Trial-based	Canada	Catheter ablation vs. antiarrhythmic drugs	3 years	2015 CDN	$34,057/QALY	(99)
Chen (2019)	Patients with an ICD and ischemic cardiomyopathy with refractory VT/VF	CUA/Markov decision model	United Kingdom	Catheter ablation vs. antiarrhythmic drugs	5 years	2018 GBP	£144,150/QALY	(100)
Calkins (2000)	Patients with an ICD and ischemic cardiomyopathy with VT/VF	CUA/Markov model	United States	Catheter ablation versus amiodarone	5 years	1998 USD	$20,923/QALY	(101)

CDN, Canadian; CUA, cost utility analysis; ICD, implantable cardioverter defibrillator; GBP, British Pound Sterling; QALY, quality adjusted life years; LV, left ventricular; VF, ventricular fibrillation; VT, ventricular tachycardia.

A trial-based economic evaluation was conducted alongside the VANISH (Ventricular Tachycardia Ablation vs. Escalated Antiarrhythmic Drug Therapy in Ischemic Heart Disease) trial, which is currently the largest trial comparing escalation of anti-arrhythmic therapy (with either amiodarone or mexiletine) to VT ablation in patients with ICDs and ischemic cardiomyopathy who failed initial anti-arrhythmic therapy (102). VANISH found that ablation was more effective at reducing the incidence of the primary composite endpoint of VT storm, death or ICD shock (HR: 0.72, 95% CI: 0.58–0.98), although there was no difference in all-cause death compared to escalated anti-arrhythmic therapy.

The economic analysis was notable for several reasons. In addition to prospective collection of health resource use to inform the economic analysis, VANISH also systematically collected health-related quality of life. From the perspective of the Canadian healthcare system, catheter ablation was economically attractive compared to antiarrhythmic therapy with an estimated ICER of $34,057 (2015 CAD) per QALY gained (99). However, the finding of cost-effectiveness varied based on the findings of a pre-specified subgroup analysis that demonstrated an interaction between baseline antiarrhythmic drug prior to trial enrollment (i.e., amiodarone or sotalol) and the primary clinical composite endpoint (102). That is, for patients who had VT refractory to amiodarone therapy, catheter ablation “dominated” escalated therapy by providing additional QALYs and a cost-savings of $769 (95% CI: −$27,092 to $35,330) over a 3-year time horizon. For sotalol-refractory VT, there was no difference in QALYs and increased total costs with ablation (99).

The well-conducted, trial-based economic evaluation of VANISH provides evidence for cost-effectiveness of ablation in drug-refractory VT particularly among the subgroup of patients with ischemic cardiomyopathy and amiodarone-refractory VT. However, there is limited data to extrapolate these economic findings to the broader patient population with VT. That is, the cost-effectiveness of VT ablation among patients with non-ischemic cardiomyopathy is unknown. Furthermore, in the context of recent trials demonstrating the effectiveness of earlier VT ablation (103–105), cost-effectiveness studies are still required to assess the value proposition of VT ablation in patients naïve to antiarrhythmic therapy. Finally, there is limited data regarding the value proposition of less complex VT ablation for other indications such as those without structural heart disease (i.e., outflow tract VT ablation, Belhassen VT), other cardiomyopathies such as arrhythmogenic cardiomyopathy, and bundle branch re-entry VT.

## Limitations of cost-effectiveness analysis

**Figure 3:**
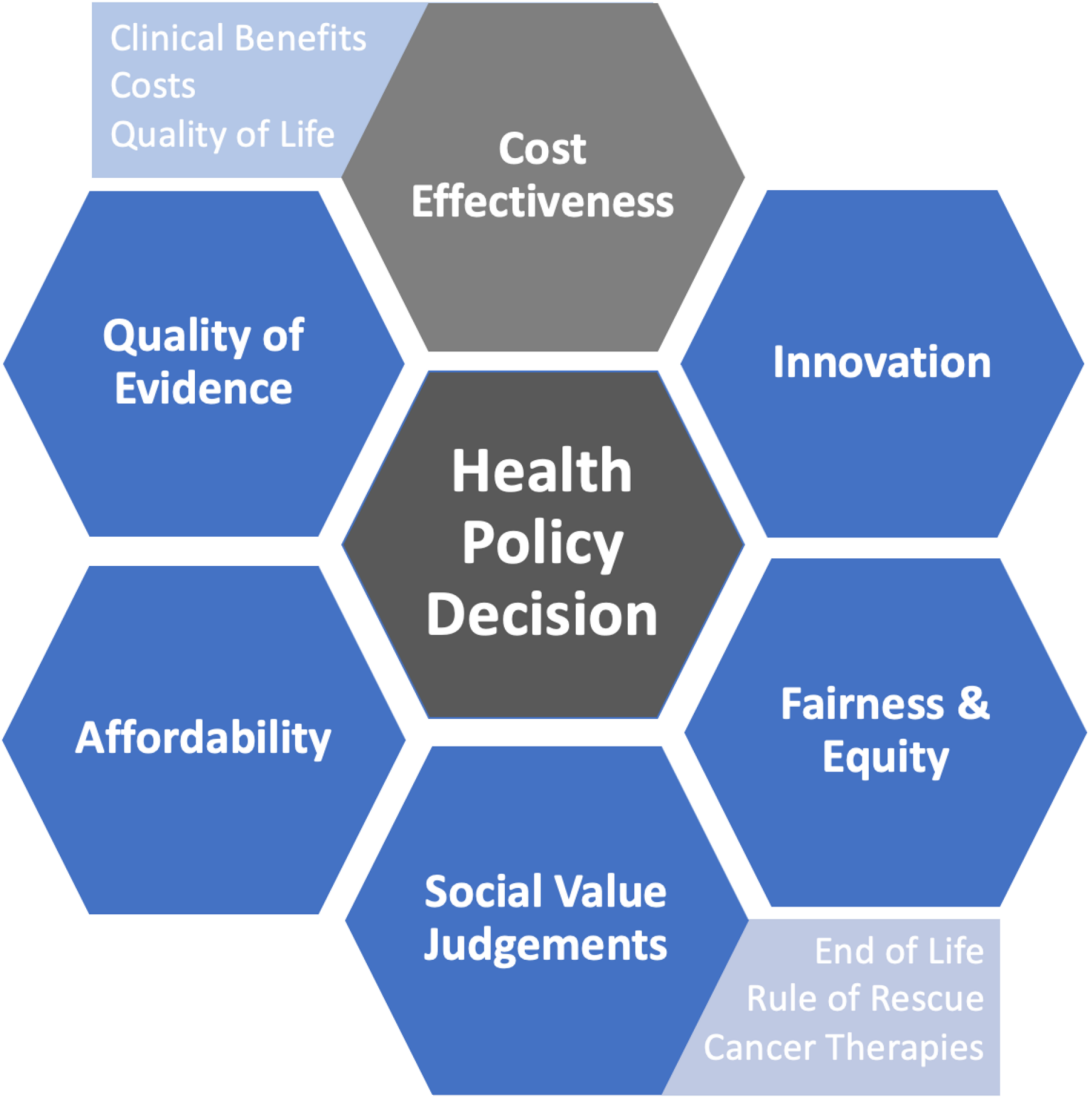
Considerations that may influence health policy decisions in addition to cost-effectiveness.

While cost-effectiveness analyses are useful tools to facilitate health policy decisions, these methods only consider costs and clinical effectiveness as key factors that inform the decision-making process. In reality, health decisions are more nuanced and influenced by additional factors (Figure 3). For example, even if adopting a new therapy is deemed cost-effective, it may not be fiscally sustainable for a given health system's finite budget due to associated opportunity costs (i.e., if a particularly large number of people are eligible for a therapy and absolute cost become exceedingly high and requiring reallocation of resources). This is especially germane to health systems which are publicly financed and those with a global budget approach to resource allocation. In addition, when considered on a global health scale, other considerations may dominate the value equation.

Societal values also are key aspects in the decision-making process. Immediately life-saving interventions (i.e., rule of rescue) and treatments for vulnerable groups (e.g., children) may be more influential than costs and clinical effectiveness alone, where there may be greater weight placed on these societal values over cost-effectiveness (106, 107). For example, an alternate funding model and process of appraisal was established for oncology therapies in England in 2010 with an update in 2016 (108). The purpose of a separate funding and appraisal mechanism was to provide patients more timely access to promising cancer therapies that would be potentially rejected on basis of conventional cost-effectiveness (108). Additionally, treatments should also be distributed equally throughout society with fair access for vulnerable and marginalized populations (109). As therapeutic options continue to expand for a number of diseases in the context of limited healthcare resources, funding decisions will continue to become increasingly complex and difficult. More comprehensive methodologies, such as multi-criteria decision analysis, are starting to gain traction among health technology agencies to help inform health decisions that take into account these additional considerations of affordability, equity, access, and other societal values (110, 111).

## Conclusion

Cardiac electrophysiology encompasses many growing technologies with important clinical applications. Many of these applications will come at a cost for increased clinical benefit. Decisions about whether or not to fund these therapies within a constrained health care budget is challenging. Cost-effectiveness analyses inform health policy decisions through the exploration of the complex relationship between costs and clinical outcomes. These analyses also assess directly the clinical benefits accrued over time from the adoption of a new technology and compare those apparent benefits to the known additional costs. In this way, an estimation of an individual intervention's value is presented for the consideration of health care policymakers. The role of formal economic evaluation is increasingly important as the rate of innovation in both drug and device development outpaces available health care expenditure. Tailored adoption of novel device and drug technology on the basis of their societal value will help facilitate a fiscally sustainable health care system.
